# Computational characterization of the peptidome in transporter associated with antigen processing (TAP)-deficient cells

**DOI:** 10.1371/journal.pone.0210583

**Published:** 2019-01-15

**Authors:** Antonio J. Martín-Galiano, Daniel López

**Affiliations:** 1 Unidad de Bioinformática, Centro Nacional de Microbiología, Instituto de Salud Carlos III, Majadahonda, Madrid, Spain; 2 Unidad de Procesamiento Antigénico, Centro Nacional de Microbiología, Instituto de Salud Carlos III, Majadahonda, Madrid, Spain; University College London, UNITED KINGDOM

## Abstract

The transporter associated with antigen processing (TAP) is a key element of the major histocompatibility complex (MHC) class I antigen processing and presentation pathway. Nonfunctional TAP complexes impair the translocation of cytosol-derived proteolytic peptides to the endoplasmic reticulum lumen. This drastic reduction in the available peptide repertoire leads to a significant decrease in MHC class I cell surface expression. Using mass spectrometry, different studies have analyzed the cellular MHC class I ligandome from TAP-deficient cells, but the analysis of the parental proteins, the source of these ligands, still deserves an in-depth analysis. In the present report, several bioinformatics protocols were applied to investigate the nature of parental proteins for the previously identified TAP-independent MHC class I ligands. Antigen processing in TAP-deficient cells mainly focused on small, abundant or highly integral transmembrane proteins of the cellular proteome. This process involved abundant proteins of the central RNA metabolism. In addition, TAP-independent ligands were preferentially cleaved from the N- and C-terminal ends with respect to the central regions of the parental proteins. The abundance of glycine, proline and aromatic residues in the C-terminal sequences from TAP-independently processed proteins allows the accessibility and specificity required for the proteolytic activities that generates the TAP-independent ligandome. This limited proteolytic activity towards a set of preferred proteins in a TAP-negative environment would therefore suffice to promote the survival of TAP-deficient individuals.

## Introduction

The proteasome, as well as other cytosolic proteases, continuously degrades misfolded or prematurely terminated proteins, also named defective ribosomal products (DRiPs), and mature proteins with normal turnover kinetics. This proteolysis generates short peptides that are transported into the endoplasmic reticulum (ER) by the transporter associated with antigen processing (TAP) [1]. In the ER lumen, the multisubunit peptide-loading complex assembles nascent MHC class I heavy chain, β_2_-microglobulin and peptides to generate trimolecular stable MHC/peptide complexes that, after export to the cell surface, are recognized by cytolytic CD8^+^ T lymphocytes (reviewed in [2]). This antigen presentation pathway is the key element in the immune response against viruses and tumors.

Mutations in the TAP genes might generate nonfunctional TAP complexes that subsequently impair the transport of cytosolic peptides to the ER, as described both in mice [3] and humans [4]. Animals and patients with this MHC class I immunodeficiency present a very limited functional CD8^+^ T cell population. Remarkably, these individuals have a limited predisposition to suffer chronic respiratory bacterial, but not viral, infections or neoplasms and they are asymptomatic for long periods. As cytotoxic CD8^+^ T cells are required to control and eliminate both malignant and virus-infected cells, their ability to recognize TAP-independent peptide antigens seems to help protect against tumor and viral infections in immunocompromised individuals.

Although TAP-independent viral epitopes were identified decades ago (reviewed in [5–7]), very few studies have analyzed the cellular TAP-independent MHC class I peptidome [8–12]. In these articles, the properties of cellular TAP-independent ligands have been defined using extensive analysis by mass spectrometry analyses. However, the nature of the parental proteins of TAP-independent ligands has remained largely unaddressed. Thus, in the present report, we applied several algorithms to perform an in-depth analysis of the features of the parental proteins for TAP-independent MHC class I ligands identified by mass spectrometry.

## Materials and methods

### Data acquisition and management

Protein descriptors and peptide coordinates were obtained from the original references. Sequences were collected using these descriptors from current versions of NCBI [13], IPI (http://ftp.ebi.ac.uk/pub/databases/IPI/last_release/) and UniProt databases [14] Peptides assigned to proteins that not were found in any of these databases (<0.5%), due to discontinuation or deletion, were not considered in the analysis. Redundant sequences present in different databases, were unified under one single descriptor using a BLAST all-against-all search of the original data and applying the threshold of 100% for both identity and alignment length. The correct positioning of the peptides in their respective protein sequences was verified in all cases, using the original manuscripts.

### Functional bioinformatics procedures

The isoelectric point and GRAVY index were calculated using the ProtParam tool of EXPASY [15]. The GRAVY index was calculated using the Kyte and Doolitle hydrophobicity scale [16]. Transmembrane helices were predicted with TMHMM 2.0 [17]. Signal peptides were predicted with SignalP 4.1 by selecting eukaryotes as the organism group [18]. Gene Ontology data were downloaded from GO web [19]. Human gene associations were downloaded from http://www.ebi.ac.uk/GOA/human_release. RNA-Seq data from the spleen were downloaded from RNA-Seq Atlas [20]. Proteins in the TAP dataset were compared to gene products in the RNA-Seq Atlas using BLAST [21], and proteins showing >95% identity and >95% length were considered identical. The human proteome was downloaded from the UniProt database (http://www.uniprot.org/proteomes/UP000005640). Proteins were associated with a given dataset (TAP+, TAP^-^C, TAP^-^NC or HLA-II) when they were strictly contained in the dataset or the ratio to the observed to expected counts, considering the dataset size with respect to the total, was greater than 2. Cleavage statistics and residue composition were assessed using in-house Perl scripts.

### Statistics

The significance of countable variables was estimated using the Chi-square test by comparing observed counts to those expected according to data from the control, TAP-sufficient cells. Likewise, differences in distributions where assessed using two-tailed Student’s t-test by comparing the non-TAP+ datasets to the control TAP-sufficient dataset.

## Results

### Data description

Several datasets were examined to investigate the study of the TAP-independent antigen processing pathways in TAP-deficient cells (Table 1). First, six datasets from four different studies, including 1051 MHC class I ligands from 727 parental proteins, were collected (Table 1). Individual studies contributed 42–543 peptides from 34–479 proteins. The TAP-deficient antigen presentation was also split into classical (TAP-C) versus nonclassical (TAP-NC) MHC, the latter was composed of the human HLA-E allele and its murine counterpart H-2 Qa-1^b^. As controls, two studies analyzing HLA class I ligands from TAP-sufficient (TAP^+^) cells (2125 peptides from 1557 proteins), including two of the alleles that were also present in the TAP-independent set (HLA-A2 and HLA-B27), were selected (Table 1). The initial data collection process was completed with six studies analyzing the HLA class II (HLA-II) peptidome (2799 peptides from 1027 proteins). Variable sensitivities and specificities should be expected due to the use of different techniques, the MHC alleles studied and the unavoidable variability of the laboratories involved. Nevertheless, the global analysis of these studies must reflect the general rules that govern the TAP-independent antigen presentation. However, the allele and study of origin were maintained throughout the manuscript to monitor such biological and technical biases. Because one of the two TAP^-^NC datasets was subjected to artificial length bias, we analyzed both datasets separately as well.

**Table 1:** Summary of datasets included in this study.

**MHC**	**TAP**	**Number of peptides**	**MHC restriction**	**Number of proteins**	**Reference**
Class I, classical	Independent	42	H-2K^d^	34	[9]
Class I, classical	Independent	112	HLA-A2	74	[10]
Class I, classical	Independent	78	HLA-B27	40	[10]
Class I, classical	Independent	196	HLA-B51 or -C1	122	[10]
Class I, non-classical	Independent	80	H-2 Qa-1^b^	70	[11]
Class I, non-classical	Independent	543	HLA-E	479	[12]
	TOTAL	1051		819	
**MHC**	**TAP**	**Number of peptides**	**MHC restriction**	**Number of proteins**	**Reference**
Class I, classical	Dependent	1545	HLA-A2	1140	[22]
Class I, classical	Dependent	580	HLA-B27	487	[23]
	TOTAL	2125		1557	
**MHC**	**TAP**	**Number of peptides**	**MHC restriction**	**Number of proteins**	**Reference**
Class II	N/A	214	HLA class II	77	[24]
Class II	N/A	612	HLA class II	321	[25]
Class II	N/A	378	HLA class II	145	[26]
Class II	N/A	448	HLA class II	143	[27]
Class II	N/A	185	HLA class II	77	[28]
Class II	N/A	962	HLA class II	264	[29]
	TOTAL	2799		1027	

**Fig 1:**
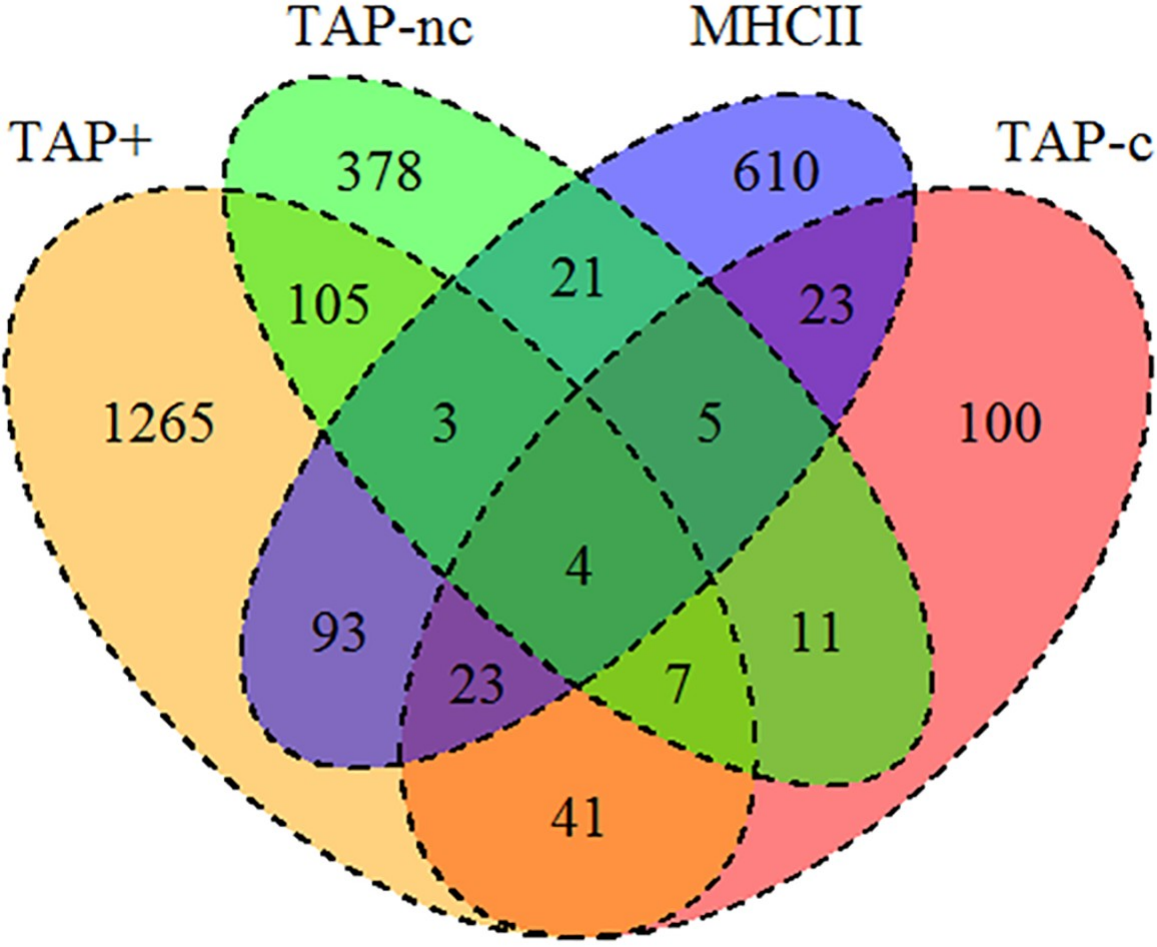
Overlap of parental proteins between the datasets included in this study. Venn diagram showing absolute numbers of proteins shared between the different datasets analyzed: TAP-sufficient MHC class I (TAP^+^), TAP-independent classical (TAP-C) and nonclassical (TAP-NC) MHC class I and MHC class II (HLA-II).

Some proteins recurrently appear in several studies, providing different peptides that bind to different alleles and are even represented in TAP-dependent, TAP-independent and HLA class II antigen processing pathways. Thus, these proteins are efficiently processed in different biochemical contexts. A relatively high overlap between TAP-independent and TAP-dependent datasets at the protein level was found (26% for the TAP-independent dataset, Fig 1). However, parental proteins from TAP-C and TAP-NC peptidomes only exhibited slight overlap (4%). Based on these data, the molecular rules governing antigen processing between TAP-C and TAP-NC should differ.

### Small and highly integral transmembrane proteins are a relevant source of TAP-C ligands

**Fig 2:**
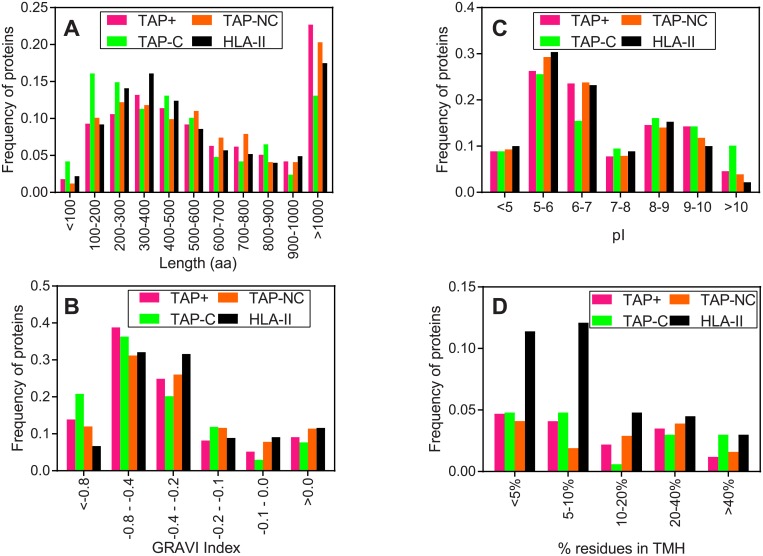
Structural features of parental proteins for MHC class I and II ligands. Protein length (panel A), isoelectric point (panel B), hydrophobicity (GRAVY index) distribution (panel C), and distribution of parental proteins according to the percentage of transmembrane residues (panel D) for the MHC peptidomes included in each dataset: TAP^+^ (magenta bars), TAP-C (green bars), TAP-NC (orange bars), and HLA-II (black bars) ligands. In panel D, only membrane proteins were considered.

Different features of the parental proteins from the four datasets were analyzed. The parental proteins for TAP^+^, TAP-NC, and HLA-II ligands had similar lengths (Fig 2A). In contrast, parental proteins for TAP-C ligands were significantly smaller than parental proteins for TAP^+^ (604 ± 667 *vs* 803 ± 877 average residues, *P* < 0.01; two-tailed Student’s t-test) and HLA class II (692 ± 708 average residues, *P* < 0.01) ligands. However, no relevant differences were observed in the pI and the hydrophobicity (Fig 2B and 2C).

In addition, the TAP^+^, TAP-C and TAP-NC datasets contained a comparable fraction of transmembrane proteins (Fig 2D). Nevertheless, parental proteins for TAP-C ligands were slightly enriched in membrane proteins with a highly integral character (>40% residues in transmembrane helices, *P* < 0.05; Chi-square analysis) compared with the TAP^+^ dataset, with a percentage similar to parental proteins for HLA-II ligands (Fig 2D). The HLA-II dataset was strongly enriched in membrane proteins, although the low percentage (<10%) of residues in transmembrane helices of most of these proteins indicates that they are peripheral rather than integral membrane proteins.

### Protein abundance is a determining factor for TAP-C presentation

**Fig 3:**
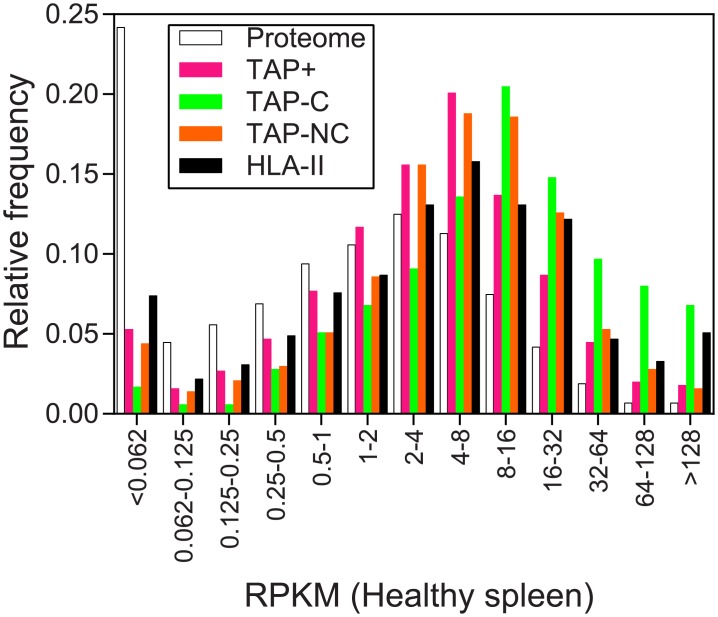
Distribution of MHC ligand-containing proteins relative to their mRNA expression levels. The expression of the parental proteins for MHC class I and II ligands and the whole proteome were depicted as RPKM (Reads Per Kilobase per Million mapped reads) of spleen mRNA levels determined using RNA-Seq. Similar to Figs 1 and 2, the four MHC ligand datasets included in this study were separately analyzed. The code color is: whole proteome (white bars), TAP^+^ (magenta bars), TAP-C (green bars), TAP-NC (orange bars), and HLA-II (black bars).

The cellular proteins encoded by mRNAs present at high concentrations are presumably abundant. Previous studies have shown that abundant proteins are likely the main source of endogenous TAP^+^ ligands, although proteins synthesized from poorly transcribed mRNAs also globally generate a significant fraction of these ligands [30–32]. Thus, considering the RNA levels of a lymphoid tissue, *i*.*e*., the spleen, as an indicator of protein levels, we confirmed that parental proteins for the TAP^+^ dataset were indeed more abundant than the average transcripts from the whole human proteome. Moreover, the link between protein abundance and MHC class I presentation was significantly stronger for the TAP-C dataset (Fig 3). In particular, proteins whose mRNAs are more abundant, measured as Reads Per Kilobase per Million mapped reads (RPKMs) > 8 were represented at a 1.94-fold higher level in the TAP-C dataset and its peptide contribution was 2.06-fold greater (*P* < 0.001; Chi-square test) than in the TAP^+^ dataset.

### Preferential processes and location of parental proteins for TAP-independent MHC class I ligands

An assay comparing protein functions between the four datasets was conducted using gene ontology enrichment. No biological processes were significantly associated with TAP^+^ ligands consistent with the fact that the proteasome evenly samples the whole proteome. However, the source of HLA-II ligands was highly enriched in genes involved in "immune and inflammatory responses", and "extracellular" or "endocytosis" processes as previously described [33] (Table 2). In addition, several central biological processes associated with mRNA production, translation and expression were linked to the TAP-C ligandome, data that correlate with their higher parental protein expression detected using the RPKM measurements (Fig 3). No biological processes were differentially associated with the generation of TAP-NC ligands. This finding, together with the low overlap between the TAP-C and TAP-NC sets showed in Fig 1, suggest that the source of these ligands seems to be different from TAP-C peptides (Table 2).

**Table 2:** Functional annotation clustering of biological process (GO term CC clustering) of parental proteins from different datasets.

GO term	Description	P-value	Detected/Expected	Dataset
0006955	immune response	4.2E-10	2.5	HLA-II
0006956	complement activation	1.6E-08	4.2	HLA-II
0002576	platelet degranulation	5.9E-08	2.6	HLA-II
0031295	T cell costimulation	7.7E-08	3.1	HLA-II
0010951	negative regulation of endopeptidase activity	1.0E-06	2.8	HLA-II
0006898	receptor-mediated endocytosis	1.2E-06	2.4	HLA-II
0008203	cholesterol metabolic process	1.3E-06	3.5	HLA-II
0030198	extracellular matrix organization	2.6E-06	2.0	HLA-II
0050852	T cell receptor signaling pathway	3.2E-06	2.7	HLA-II
0007186	G-protein coupled receptor signaling pathway	5.3E-06	2.4	HLA-II
0001895	retina homeostasis	7.0E-06	3.2	HLA-II
0050776	regulation of immune response	7.3E-06	2.2	HLA-II
0006954	inflammatory response	2.1E-05	2.2	HLA-II
0022617	extracellular matrix disassembly	3.5E-05	2.4	HLA-II
0006414	translational elongation	8.3E-09	3.7	TAP^-^C
0044267	cellular protein metabolic process	6.9E-08	2.4	TAP^-^C
0008380	RNA splicing	1.9E-06	2.9	TAP^-^C
0006412	translation	4.5E-06	2.7	TAP^-^C
0006415	translational termination	6.0E-06	3.3	TAP^-^C
0006413	translational initiation	9.5E-06	3.0	TAP^-^C
0006614	SRP-dependent targeting to membrane	1.1E-05	3.1	TAP^-^C
0019083	viral transcription	2.2E-05	3.0	TAP^-^C
0019058	viral life cycle	3.2E-05	2.8	TAP^-^C
0045892	negative regulation of transcription, DNA-templated	1.2E-04	2.9	TAP^-^C
0000184	nuclear-transcribed mRNA catabolic process	1.3E-04	2.8	TAP^-^C
0006397	mRNA processing	1.5E-04	3.0	TAP^-^C
0006457	protein folding	1.5E-04	3.0	TAP^-^C
0000398	mRNA splicing, via spliceosome	3.2E-04	2.5	TAP^-^C

In addition, cellular components of the four datasets were analyzed (Table 3). No significant cellular components were associated with the TAP^+^ ligandome, which was also consistent with the ubiquitous protein degradation by the proteasome mentioned above. On the other hand, HLA-II ligands were associated with proteins involved in the endocytic pathway (endoplasmic reticulum, Golgi, vesicles, cell membrane, and extracellular matrix), as described in previous studies [34]. The sources of TAP-C ligands were enriched in clusters associated with RNA primary transcription, ribosome or aggregation of proteins and RNAs that appear when the cell is under stress, such as cytoplasmic stress granules (Table 3). Moreover, the cellular components associated with the TAP-NC ligandome were enriched in DNA-related structures, such as the kinetochore and chromatin. Altogether, these data support the existence of newly differentiated MHC class I antigen processing pathways and compartmentalization to yield classical and nonclassical TAP-independent ligands.

**Table 3:** Functional annotation clustering of cellular components (GO term CC clustering) of parental proteins from different datasets.

GO term	Description	P-value	Detected/Expected	Dataset
0005615	extracellular space	1.2E-28	2.5	HLA-II
0005576	extracellular region	6.2E-26	2.5	HLA-II
0072562	blood microparticle	8.1E-17	3.1	HLA-II
0009897	external side of plasma membrane	2.4E-12	3.1	HLA-II
0005578	proteinaceous extracellular matrix	2.0E-10	3.3	HLA-II
0005765	lysosomal membrane	2.1E-08	2.3	HLA-II
0042613	MHC class II protein complex	3.4E-08	3.8	HLA-II
0031012	extracellular matrix	1.0E-07	2.7	HLA-II
0032588	trans-Golgi network membrane	3.5E-07	3.2	HLA-II
0012507	ER to Golgi transport vesicle membrane	8.2E-07	2.4	HLA-II
0071556	integral component of lumenal side of ER	1.2E-06	2.4	HLA-II
0031093	platelet alpha granule lumen	6.7E-06	3.0	HLA-II
0042627	chylomicron	1.2E-05	4.1	HLA-II
0034361	very-low-density lipoprotein particle	2.4E-05	3.6	HLA-II
0030658	transport vesicle membrane	2.9E-05	2.9	HLA-II
0034362	low-density lipoprotein particle	6.4E-05	4.1	HLA-II
0034366	spherical high-density lipoprotein particle	6.4E-05	4.1	HLA-II
0071682	endocytic vesicle lumen	6.4E-05	4.1	HLA-II
0030666	endocytic vesicle membrane	7.6E-05	2.7	HLA-II
0030669	clathrin-coated endocytic vesicle membrane	9.8E-05	2.7	HLA-II
0030139	endocytic vesicle	1.4E-04	3.0	HLA-II
0043202	lysosomal lumen	1.5E-04	2.5	HLA-II
0034364	high-density lipoprotein particle	6.2E-04	3.4	HLA-II
0016324	apical plasma membrane	9.4E-04	2.1	HLA-II
0030529	ribonucleoprotein complex	1.3E-10	4.3	TAP^-^C
0005689	U12-type spliceosomal complex	3.8E-07	6.6	TAP^-^C
0022627	cytosolic small ribosomal subunit	1.8E-06	4.4	TAP^-^C
0005925	focal adhesion	5.6E-06	2.4	TAP^-^C
0010494	cytoplasmic stress granule	7.8E-06	5.5	TAP^-^C
0005903	brush border	1.5E-05	4.6	TAP^-^C
0071013	catalytic step 2 spliceosome	4.8E-05	3.4	TAP^-^C
0005681	spliceosomal complex	1.1E-04	3.7	TAP^-^C
0000776	kinetochore	6.4E-05	2.8	TAP^-^NC
0005832	chaperonin-containing T-complex	8.2E-05	4.2	TAP^-^NC
0000785	chromatin	3.4E-04	2.3	TAP^-^NC
0002199	zona pellucida receptor complex	5.4E-04	3.6	TAP^-^NC
0000922	spindle pole	6.3E-04	2.4	TAP^-^NC

### TAP-independent ligands were predominantly derived from the N- and, to a greater extent, C-terminal regions of parental proteins

**Fig 4:**
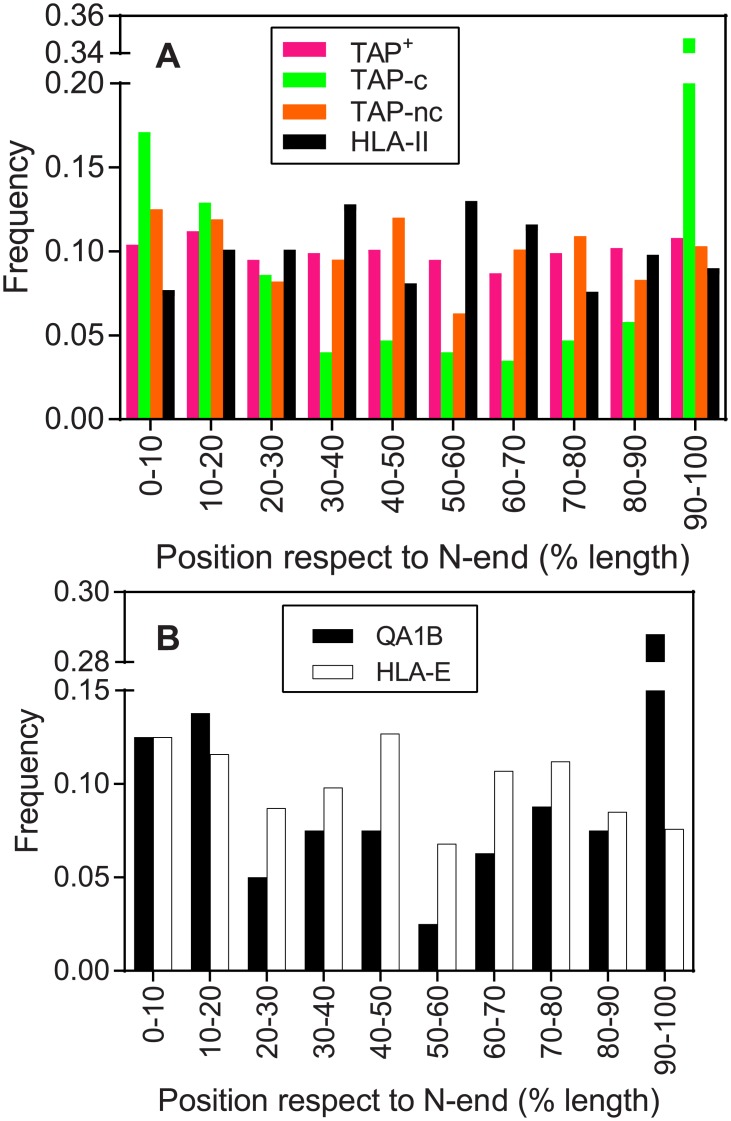
Positions of MHC ligands in parental proteins. Relative positions relative to the protein N-terminus for TAP^+^ (magenta bars), TAP-C (green bars), TAP-NC (orange bars), and HLA-II (black bars) ligands (panel A). Panel B: Murine (black bars) and human (white bars) MHC alleles in TAP-NC were analyzed separately.

TAP^+^ ligands were evenly distributed over the sequence of respective parental proteins (Fig 4A), indicating that the antigen processing by proteasomes mainly occurs on fully denaturalized proteins, as was classically described. Reversely, when the position of the TAP-C ligandome was mapped on the respective parental proteins whose lengths have been split into deciles, a “smile-shaped” graph was obtained. Specifically, the two N-terminal and the C-terminal deciles were overrepresented with respect the findings expected for random distribution, with an even more predominant contribution of the later category (Fig 4A). Although the TAP-NC ligandome exhibited a similar random distribution to TAP^+^ ligands (Fig 4A), when mouse and human HLA non classical alleles were separated, H-2 Qa-1^b^ (but not HLA-E) also showed a “smile-shaped” graph (Fig 4B). This finding likely indicates a different antigen processing pathway for these HLA-E ligands compared with other TAP-independent ligands bound to different classical human and nonclassical mouse MHC class I alleles, although an artificial bias due to the exclusion of longer peptides in the original study cannot be excluded. In addition, 21% of TAP-independent peptides were located exactly at the C-terminal position of their respective proteins. In these cases, only one endoproteolytic cleavage event was needed to release these particular ligands. In contrast, exact N-terminal ligands generally were not identified (less of 1% of peptides), indicating that most ligands required two different endoproteolytic cleavage events.

### The C-terminal sequences of parental proteins for HLA^-^C ligands are enriched in Gly, Pro and aromatic residues

**Fig 5:**
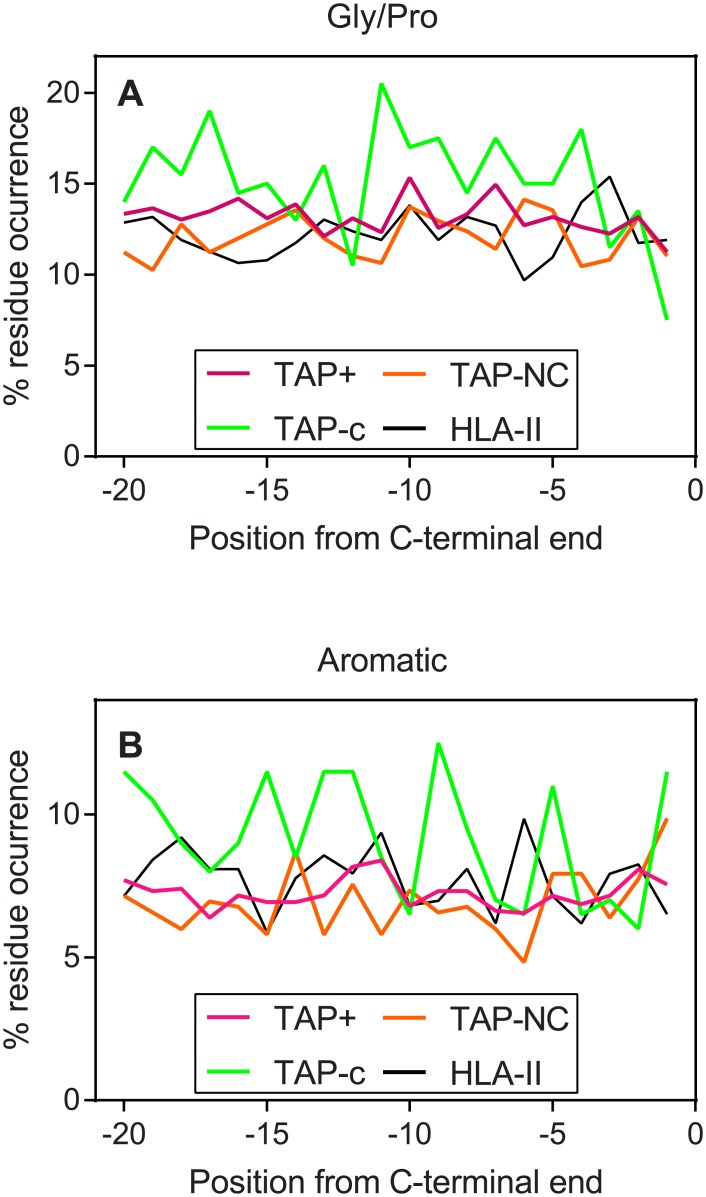
Relative residue occurrence in the C-terminus of parental proteins. Frequency of glycine and proline (panel A) and aromatic (panel B) residues in the twenty C-terminal residues of parental proteins, where the first residue is the last C-terminal residue in the sequence. The code color is in the same as described in Fig 4.

The residue composition of the last 20 positions from the C-terminus of protein was analyzed and compared with the TAP^+^ dataset to rationalize the abundance of TAP-C peptides in the C-terminal decile (Fig 4). No differences were observed with the exception of aromatic amino acids, Gly and Pro residues, which were statistically significant different despite the inherent noise (Fig 5). The two latter residues are common at the corner of turns in protein folds, which were explicitly overrepresented in several of these positions in the TAP-C dataset (Fig 5A). This effect was not observed in the extreme N-terminal, except for a Gly enrichment between positions 12 and 34 (average of 10.2% vs 8.7% in the TAP+ control; P-value: 0.002; Student’s t -test).

### Cleavage specificity of peptidases involved in the generation of TAP-independent ligands

**Fig 6:**
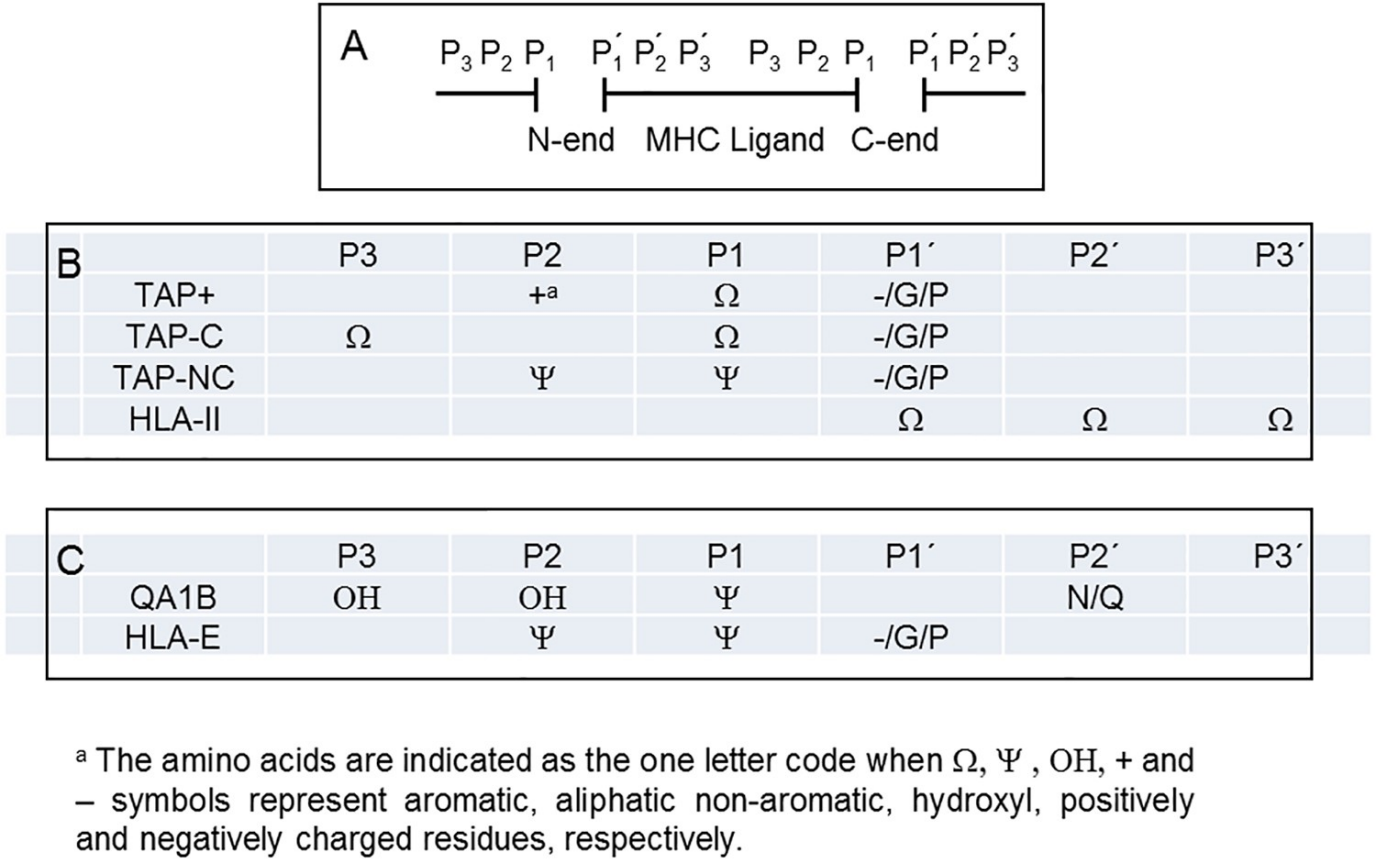
Analysis of N- and C-terminal cleavage specificity in MHC ligands. A diagram of residues involved in the generation of naturally processed MHC ligands by peptidase cleavage is shown (panel A). Distribution of P_1_ to P_3_ and P´_**1**_ to P´_**3**_ amino acid residues of the scissile bonds created by peptidase cleavage in the four datasets of (panel B) murine (black bars) and human (white bars) MHC allele in TAP-NC were separately analyzed (panel C). Only residues displaying 2-fold enriched with respect to the expected value and with p-values < 0.001 were considered.

Finally, the specificity of peptidases involved in generating ligands in TAP-defective cells was addressed by determining the distribution of individual amino acid at the three flanking residues (P_3_, P_2_, P_1_, P_1_´, P_2_´ and P_3_´) on both sides of the hydrolyzed bonds of MHC ligands. Absolute residue counts at each position were normalized using the natural residue occurrence in the selected proteins. Fig 6 shows the distribution of amino acids, clustered by biochemical groups, found in the different flanking positions of scissile bonds.

The four datasets showed amino acids enrichment at several positions of flanking residues. The specificity of peptidases involved in the generation of TAP-independent ligands was more similar to peptidases that generate the TAP^+^ peptides than enzymes involved in generating HLA-II antigens. However, as shown in Fig 6, differential enrichment of aromatic residues at the P3 position of the hydrolyzed bonds in the TAP-C ligandome and aliphatic residues enrichment at the P1 and P2 positions in the TAP-NC peptidome indicates that proteases with different specificity are involved in classical MHC class I antigen presentation generating the classical and nonclassical peptide pools. Additionally, mouse and human HLA non classical alleles were separately analyzed, and differential enrichments of hydroxyl and amide (N/Q) residues was observed at the P3, P2 and P2´ positions of parental murine proteins.

## Discussion

In this study, we provide information on the features of parental proteins, the source of TAP-independent ligands presented by alternative antigen processing pathways to the classical proteasome, TAP-dependent pathway. These results may explain why TAP mutations do not result in a lethal phenotype [3,4]. A systemic computational approach was used in the present study.

Several thousand ligands bound to specific MHC class I alleles were identified in an immunoproteomic analysis of TAP-sufficient cells in the previous studies [22,23]. In contrast, in the small number of similar studies examining TAP-deficient cells only tens [8,9] [11] or hundreds [10,12] of TAP-independent MHC peptides have been described. This finding is consistent with the very low expression of MHC class I molecules on the surface of TAP-deficient cells compared with normal, TAP-sufficient cells. The main difference between TAP-dependent and -independent MHC ligandomes analyzed using mass spectrometry in previous studies was the increased peptide lengths and the absence of strict binding motifs in the latter. In the later years, spliced peptides derived from a transpeptidation reaction mediates by proteasome activity between fragments distant in the parental protein have been described [35,36]. Although these these peptides are also longer than the TAP-dependent ligands, their contributions to antigen processing in TAP-deficient cells are very limited [37]. These differences between TAP-dependent and TAP-independent MHC ligandomes applied to all MHC class I alleles studied. The exception was the study by Weinzierl et al. [8], in which, in contrast to other TAP-independent peptidomes analyzed, the ligands were theoretically assigned to the respective HLA class I molecules using two in silico web tools: BIMAS (https://www-bimas.cit.nih.gov/) and SYFPEITHI (http://www.syfpeithi.de/) that only predict 8, 9 or 10 mer high affinity ligands. Thus, longer peptides or ligands with a low predicted HLA binding score (the majority of TAP-independent ligands in other mass spectrometry analyses performed [9,10]) were not detected by these algorithms and thus, the conclusions might be biased towards a rather limited nonrepresentative subset of the total TAP-independent HLA peptidome. Therefore, this study was not included in the present report. On the other hand, the data reported by Lampen *et al*. present an intermediate situation, since no bioinformatics strategy was used but only 8–13 mer peptides were manually included in the analysis of the nonclassical HLA-E peptidome [12]. Thus, in this mass spectrometry analysis, low affinity ligands were detected, but the most striking TAP-independent ligands (whose length is even greater than 20 residues) were excluded. This study was included in the current report, but independently analyzed to detect possible sources of bias as these peptides were more hydrophobic. In addition, as the same laboratory had previously analyzed the TAP-independent ligandome of murine orthologue (H-2 Qa-1^b^) of HLA-E with the same mass spectrometer and experimental procedures without length restriction [11], the effect of length selection was studied. The analysis of features of the proteins and peptides included in this report indicated that murine H-2 Qa-1^b^ and human HLA-E peptidomes differed, as TAP-independent H-2 Qa-1^b^ ligands exhibited very similar characteristics to TAP-C ligandomes from either murine or human TAP-deficient cells. Therefore, dramatic differences do not exist between the antigen processing pathways for classical or nonclassical TAP-independent ligands. Thus, the MHC peptidomes should be preferentially analyzed without any methodological restriction in order to avoid reaching spurious conclusions.

The prevalence of signal sequence-derived ligands was described in early mass spectrometry analyses [38] [39] and in other studies, focused on the most abundant cellular peptides bound to some HLA class I molecules [8] from TAP-deficient cells. This fact correlates with the binding specificity of the MHC class I molecules analyzed, HLA-A2 or -B51, which bind relatively hydrophobic peptides. In contrast, for other MHC class I molecules with positively or negatively charged residues as anchor motifs, the contribution of this pathway must be minor or even residual because signal sequences do not tolerate theses residues. This finding is consistent with the origin of the viral TAP-independent epitopes identified by analyzing T cell immune responses. In a study of several MHC class I molecules, only 19% of TAP-independent viral epitopes were derived from signal sequences, a value that was less than those derived from other sources: luminal, transmembrane or even cytosolic proteins [40]. For example, only 2 of 13 TAP-independent ligands naturally presented by six different HLA class I molecules from vaccinia virus-infected TAP-deficient cells were derived from signal sequences [41]. Moreover, these low percentages may be overestimated, since HLA-A2 is the most frequently studied allele in antiviral immune responses.

Cellular proteins derived from highly abundant mRNA are much more common as a source of the TAP-independent ligandome than TAP-dependent HLA class I ligands. In addition, biological processes associated with mRNA production, translation and expression were also linked to the TAP-C ligandome. Both correlate with the poor performance of alternative antigen processing pathways compared with the high efficiency of classical proteasome, -TAP-dependent pathway. Thus, a reasonable hypothesis is that the protein abundance simply increases the possibility of interaction with the different "nonprofessional" antigen-producing proteases residing in the cellular compartments associated with TAP-independent antigen processing pathways in a TAP-free environment. Although the peptide and mRNA data were acquired from different sources, i.e., proteomic peptides from lymphoblastoid cell lines and RNA-Seq data from the spleen, respectively, these are more biologically relevant in the natural environment of the later. Nevertheless, B cells are, by far, the predominant cellular type in the mouse spleen. Therefore, a worst case explanation is our results are an underestimation of the protein abundance as a key factor in TAP-deficient cells.

A strong tendency of TAP-C and H-2 Qa-1^b^ to bind peptides located at the ends of the parental protein was observed. Notably, 30% and 35% of the TAP-C ligandome were present in the first two N- and the C-terminal sequence deciles, respectively. Similarly, 55% of H-2 Qa-1^b^ ligands were located in these regions. Unlike the proteasome, when the protein substrates are unfolded by the 19S regulatory particle prior to their degradation, the peptidases that operate in the TAP-independent pathways must act on folded proteins. Only the surface of these proteins would be exposed, even transiently, and susceptible to protease activity, which reduces the diversity of potential ligands. These TAP-independent ligands were usually located at the exact C-terminal position of their respective proteins. The underlying explanation for this finding may be that peptides are released by a single cleavage event within the fully translated, likely folded protein which would favor the generation of ligands in a proteolysis-poor context. In addition, we identified a relative over-occurrence of Gly and Pro residues in these C-terminal regions. As Gly provides high flexibility to polypeptide chains, this amino acid is frequently found in protein regions without secondary structures, such as loops or coils. Pro is a structure-disrupting residue that is often found in loop regions. Altogether, this combination might explain, in terms of accessibility to proteolytic targets, the preference of TAP-independent antigen processing pathways for these C-terminal regions.

On the other hand, TAP-C or H-2 Qa-1^b^ N-terminal peptides rarely include the first residue itself and only Gly residue biases were observed in this terminal region. A plausible explanation for the high N-terminal preference is the existence of nascent proteins that have not reached the critical number of translated residues to trigger the folding process, and thus proteases have a greater chance of targeting these sequences in this temporal window. In support of this hypothesis, two of the three most significant enriched cellular component GO terms (P-value < 10^−4^ compared to TAP^+^ data) for unique TAP-C proteins with peptides located in the first 50 positions of the N-terminus were "extracellular exosome" and "membrane". These proteins are initially cotranslationally inserted to the ER and thus, cleavages of their nascent unfolded DRiPs would generate TAP-independent ligands for MHC binding.

A relative abundance of aromatic or aliphatic residues in different flanking positions of scissile bonds and, for the Phe/Trp/Tyr amino acids, in the neighborhood of C-terminal regions of parental proteins, was observed in regions that are preferential source of TAP-independent ligands. Based in this finding, endoproteolytic proteases with specificity for these amino acids are likely relevant in the antigen processing pathway in TAP-deficient cells. Among these enzymes, cathepsins (MEROPS database: http://merops.sanger.ac.uk [42]), which were previously shown to involved in HLA class II antigen processing [43], show aromatic or aliphatic cleavage specificities and would be relevant candidates for generation of the TAP-independent ligands with adequate flanking regions.

Fundamental cellular processes, such as peptide presentation, rely on the robustness of proteins networks and therefore they must be explored with the lens of systems biology. Thus, antigen processing and presentation tolerates the absence of TAP by recruiting nonspecialized proteases that act as a safety net when the involvement of the proteasome is limited. These secondary alternative pathways contribute residually to total antigen processing in TAP-sufficient cells as assessed using TAP knockout-cell lines. Te safety range for peptide presentation is substantially reduced but still significantly active in these cells. This observation represents the sum of marginal advantages that altogether counterbalance the lack of a dedicated proteolytic machinery such as the proteasome. This collective alternative appears to be sufficient to sustain the immunological clearance of most infections in TAP-deficient individuals.

In summary, the global picture emerging from the current report suggests that the TAP-independent antigen processing pathways are preferentially focused on small, abundant proteins with numerous Gly, Pro and aromatic residues in their C-termini, favoring peptide generation through a single cleavage event. Both the still-unfolded N-terminal and unfolded C-terminal sequences of parental proteins allow the protease to access and specifically perform the proteolytic cleavages that mainly generate the TAP-independent peptidome.
